# Predicting smoking cessation, reduction and relapse six months after using the Stop-Tabac app for smartphones: a machine learning analysis

**DOI:** 10.1186/s12889-023-15859-6

**Published:** 2023-06-05

**Authors:** Jean-François Etter, Germano Vera Cruz, Yasser Khazaal

**Affiliations:** 1 Institute of Global Health, Faculty of Medicine, University of Geneva, Geneva, Switzerland; 2 Department of Psychology, UR 7273 CRP-CPO, University of Picardie Jules Verne, Chemin du Thil, Amiens, 80025 France; 3 Addiction Medicine, Lausanne University Hospital, Lausanne, Switzerland; 4 Lausanne University, Lausanne, Switzerland; 5 Department of Psychiatry and Addictology, Montreal University, Montreal, Canada; 6 Département de Psychiatrie, Service de médecine des addictions, Lausanne University Hospital, Rue du Bugnon 23, Lausanne, 1011 Switzerland

**Keywords:** Smartphone apps, Smoking reduction, Smoking cessation, Smoking relapse

## Abstract

**Background:**

An analysis of predictors of smoking behaviour among users of smoking cessation apps can provide useful information beyond what is already known about predictors in other contexts. Therefore, the aim of the present study was to identify the best predictors of smoking cessation, smoking reduction and relapse six months after starting to use the smartphone app Stop-Tabac.

**Method:**

Secondary analysis of 5293 daily smokers from Switzerland and France who participated in a randomised trial testing the effectiveness of this app in 2020, with follow-up at one and six months. Machine learning algorithms were used to analyse the data. The analyses for smoking cessation included only the 1407 participants who responded after six months; the analysis for smoking reduction included only the 673 smokers at 6-month follow-up; and the analysis for relapse at 6 months included only the 502 individuals who had quit smoking after one month.

**Results:**

Smoking cessation after 6 months was predicted by the following factors (in this order): tobacco dependence, motivation to quit smoking, frequency of app use and its perceived usefulness, and nicotine medication use. Among those who were still smoking at follow-up, reduction in cigarettes/day was predicted by tobacco dependence, nicotine medication use, frequency of app use and its perceived usefulness, and e-cigarette use. Among those who had quit smoking after one month, relapse after six months was predicted by intention to quit, frequency of app use, perceived usefulness of the app, level of dependence and nicotine medication use.

**Conclusion:**

Using machine learning algorithms, we identified independent predictors of smoking cessation, smoking reduction and relapse. Studies on the predictors of smoking behavior among users of smoking cessation apps may provide useful insights for the future development of these apps and future experimental studies.

**Clinical trial registration:**

ISRCTN Registry: ISRCTN11318024, 17 May 2018. http://www.isrctn.com/ISRCTN11318024.

## Background

Smartphone applications (apps) for smoking cessation provide timely information, feedback, individually tailored counseling, follow-up for several months, and can reach large numbers of smokers at a low cost per participant [1]. Some of these apps slightly increase smoking cessation rates compared to control conditions [1]. In order to develop and improve smoking cessation apps, it is important to determine which categories of users benefit most, but also which types of smokers benefit less and therefore require either additional in-app support (e.g., specific emotion regulation tools or transdiagnostic targets) or require human support (e.g., telephone or in-person) [2, 3]. An analysis of predictors of smoking cessation, smoking reduction and relapse among users of smoking cessation apps may provide useful information beyond what is already known about predictors identified in other contexts. Examples of such predictors include motivation, dependence level, cravings and other withdrawal symptoms, duration of prior attempts to quit smoking, social and professional support, smokers in the household, among friends and colleagues, self-efficacy, attitudes toward smoking cessation, use of self-change strategies, use of medications or other nicotine-containing products, mental health, negative affect, social support, alcohol use, and drug use [4–6].

### Study purpose

The present study is a secondary analysis of data from a randomized trial testing the efficacy of the Stop-Tabac smartphone app for smoking cessation [7]. The current study aims to identify the best predictors of smoking cessation, smoking reduction and smoking relapse six months after starting the intervention. This study also aimed to examine the relationships (probability of association) between the predictors or covariates and smoking cessation, smoking reduction and smoking relapse at 6 months.

### Research questions

We examined six questions: what are the best predictors (in rank order) of:

#### (RQ1):

Smoking cessation after six months,

#### (RQ3):

Change in the number of cigarettes/day between baseline and 6 months in those who continued to smoke after six months,

#### (RQ5):

Relapse to smoking after six months, in participants who stopped smoking after one month.

And what are the relationships (probabilities of association) between the predictors or covariates and:

#### (RQ2):

Smoking cessation after six months,

#### (RQ4):

Change in number of cigarettes/day between baseline and six months,

#### (RQ6):

Relapse to smoking after six months.

## Methods

### Participants

We enrolled 5293 daily smokers in the randomized trial. The one-month follow-up questionnaire was completed by 1861 participants (35% of 5293), and the six-month follow-up questionnaire was completed by 1407 participants (27% of 5293). Only the 1407 participants who answered the six months questionnaire were included in the analyses of smoking cessation. Only the 673 participants who were still smoking after six months and provided information on cig./day both at baseline and at 6 months were included in the analyses of change over time in cig./day. Only the 502 participants who had quit smoking after one month (criterion: no smoking in the last 7 days) and who responded after 6 months were included in the analyses on relapse.

### Recruitment and sampling procedures

The recruitment process has already been described in detail [7, 8]. In brief, eligible participants were adult daily smokers who lived in Switzerland or France, had decided to quit smoking, and had set a quit date within one month before enrolment in the study. Participants were recruited in 2019–2020 via advertisements on the internet and registered online. After downloading the app for free from the Apple App store or Google Play store, they clicked on a link that led to an online consent form and an online screening and baseline questionnaire. Eligible participants were then randomly assigned to receiving either a code to unlock a full version of the Stop Tabac smoking cessation app or a code to unlock a control version of the app that contained only a few features (both versions were in French). The eligibility assessment was automatic. The full version of the Stop-tabac app includes information pages, calculators (number of cigarettes not smoked, days of life gained and money saved since quitting smoking), customised (automated) counselling reports, a quiz, a discussion forum, telephone numbers of quitlines, and an interactive module on nicotine replacement therapy and e-cigarettes. The app was a stand-alone intervention and there was no human involvement in this smoking cessation intervention. The full version of the Stop-tabac app is available for free from the Apple App store and the Google Play store. The control version contained the above calculators and 5 pages of 100–300 words each on the addictive potential of nicotine, reasons to quit and money savings.

### Data collection

Participants were asked by email to answer short follow-up questionnaires online after one and six months. Those who did not respond were reminded several times by e-mail, then by Whatsapp or text messages (SMS), then by post and then by phone call. The enrolment and consent form and the questionnaires for the initial and follow-up surveys are available here:

https://archive.org/details/@stopdependance_ch.

#### Predictors and covariates

Predictor variables were measured at baseline and after one month and were used to predict smoking cessation or relapse and cigarette consumption after six months. Covariates were measured at six months to examine whether they were associated with smoking status and cigarette consumption at six months. Only predictor variables were used in the machine learning models, but both predictors and covariates were used in the multinomial logistic regression models. At the beginning of the study, the following predictor variables were collected: country of residence, age, gender, a 2-item screening test for depression (yes, no) [9], experimental group (full Stop-Tabac app or control app), number of years as a smoker, number of cigarettes smoked daily, number of minutes between waking up and smoking the first cigarette of the day (an indicator of tobacco dependence) [10]), current smoking of other tobacco products, current use of heated tobacco and electronic cigarettes (the latter three variables with 4-point response options), and current use of nicotine replacement products.

Predictors measured at one month included smoking status (no tobacco use in the past 7 days and in the past 4 weeks), number of cigarettes smoked per day, minutes to first cigarette of the day, quit attempts after entering the study (yes/no), intention to quit smoking (3-point response scale), use of nicotine medication, e-cigarettes or heated tobacco after entering the study (yes, no), self-reported use of a smoking cessation app in the past month (yes, no) and perceived usefulness of the study app (6-point scale).

Covariates and outcomes measured at six months included the same variables measured at one month, plus three variables automatically collected by the app from all participants: the number of different days the app was accessed, the number of times the app was opened, and the duration of app use, i.e. the interval in days between the first and last day participants used the app.

For predictors, we did not include smoking status and cigarettes/day after one month, and for covariates, we did not include cigarettes/day after six months, as these variables captured most of the variance as they were very similar to the outcome variables.

#### The outcome variables

The three outcome variables were: self-report of not having smoked in the past four weeks assessed at six months (yes = 1, no = 0, in 1407 participants), change in daily cigarette consumption between baseline and six months (only among those who were still smoking and reported cigarettes/day at six months, 673 participants), and relapse to smoking at six months (yes = 1, no = 0, in 502 participants who had stopped smoking at one month). Change in cigarettes/day is a continuous variable calculated as follows: cigarettes/day after six months minus cigarettes/day at the beginning of the study, a negative value means a decrease in cigarettes/day.

### Ethics

The study protocol was submitted to the Cantonal Ethics Committee in Geneva (Req-2018-00356), which replied that the app was not a medical device and therefore the study did not need to be approved. The commission therefore did not review the protocol, but wrote in an e-mail dated 16 May 2018 that: “All indications are that this study is being conducted in accordance with the general ethical principles that apply to all research involving human subjects”. The online consent form described the study, risks and benefits, how confidentiality was maintained, and explained the data collection and data sharing procedures. Participants’ information was kept confidential and accessible only to the members of the research team (the project leaders, a computer expert and two research assistants). The dataset for analysis was anonymized, the only person who could access the code linking participants’ names and their responses to questionnaires was a computer expert under the supervision of the first author. There was no risk associated with the app itself and with the data collection procedure. We used appropriate security measures to protect the data. Participants in the control group were able to access to full Stop-Tabac app after the end of data collection. Results were published [7]. The original data set (anonymized) is available publicly at Yareta.unige.ch.

### Data analysis

First, we compared respondents to the 6-month questionnaire (n = 1407) and non-respondents (n = 3886, using chi-square tests for proportions and Mann-Whitney U tests for means) (Table 1).

**Table 1:** Baseline characteristics of respondents and non-respondents to the 6-month survey, and their utilization of the app after 6 months

	Responded at 6 months	Did not respond at 6 months
N	1407	3886
Age, years, median	38	33 ***
Sex, men, %	33	35
Country, France, %	71	86 ***
Smokes cigars, cigarillos, pipe or cannabis daily, %	16	19 *
Ever used heated tobacco, %	7	5 *
Currently uses electronic cigarettes, %	18	18
Currently uses nicotine medication, %	19	17
Depression screening test positive, %	59	65 ***
Cigarettes per day, median	15	15 **
Minutes to first cigarette of the day, median	30	20 ***
Years since started to smoke, median	19	14 ***
*Data collected automatically by the app after 6 months*:		
Number of different days when the used the app, median	10	3 ***
Number of times the opened the app	42	15 ***
Interval between first and last access to the app, days, median	67	8 ***

p-values: * p < .05, ** p < .01, *** p < .001

To answer question 1 (i.e. predictors for smoking cessation), we built a machine learning model using the random forest (RF) classification algorithm [11, 12], with the following setting parameters: “ntree” = 500, meaning that each model RF was constructed from 500 regression trees, and “mtry” = 6, meaning that the number of predictors available for partitioning at each tree node was set to 6. RF classification models provide, among other results, the importance of each predictor based on a measure called Mean Decrease Accuracy (MDA). The MDA expresses how much accuracy the model loses by excluding each variable. The more accuracy suffers, the more important the variable is for successful prediction (Table 2).

**Table 2:** Predictors of smoking cessation after six months in decreasing order of importance, in users of a smartphone app for smoking cessation, 2019–2021

Predictors	MDA
Number of different days when the app was opened (automatically collected data)	20.22
Intention to quit smoking as measured after one month	16.87
App’s use duration (automatically collected)	14.33
Number of times the app was opened (automatically collected)	12.95
Cigarettes/day as measured at baseline	8.74
Current use of any smoking cessation app, as self-reported after one month	7.83
Perceived helpfulness of the app after one month	6.12
Age	5.11
Use heated tobacco products at baseline	4.69
Number of years smoking	3.71
Minutes before the first cigarette of the day	3.18
Sex	2.97
Use of e-cigarettes after one month	2.31
Being depressed	2.24
Smoking other tobacco products at baseline	1.93
Country (Switzerland vs. France)	1.22
Use of nicotine medications after one month	0.86
Experimental group	0.77
Use of e-cigarette at baseline	0.49
Used of heated tobacco after one month	0.45

MDA = mean decrease accuracy, a statistical measure indicating the level of the predictor variable importance in the classification machine learning algorithm

To answer question 2 (i.e. relationship between each predictor and smoking cessation and association between each covariate and smoking cessation), we built a multinomial logistic regression model using SPSS software (version 28.0) (Table 3).

**Table 3:** Predictors and covariates of smoking cessation after six months, from two multivariate logistic regression models, in users of a smartphone app for smoking cessation, 2019–2021

Predictors category	Predictors / covariates	*b*	OR	SE	*p*	95%CI on OR	
MODEL 1: **Predictors measured at baseline and after one month**							
Demographics	Country	0.110	1.11	0.159	0.489	0.81	1.52
	Age	0.018	1.01	0.018	0.311	0.98	1.05
	Sex	− 0.160	0.85	0.149	0.283	0.63	1.14
Treat/Control	Experiment group	− 0.249	0.78	0.148	0.092	0.58	1.04
Baseline	Number of years smoking	− 0.021	0.97	0.009	0.022	0.96	0.99
	Cigarettes/day	0.043	1.04	0.013	< 0.001	1.01	1.07
	Minutes before the first cigarette of the day	− 0.258	0.77	0.075	< 0.001	0.66	0.89
	Use other tobacco products	0.229	1.25	0.063	< 0.001	1.11	1.42
	Use heated tobacco	− 0.225	0.79	0.314	0.473	0.43	1.47
	Use e-cigarettes	0.076	1.07	0.077	0.324	0.92	1.25
	Use nicotine medications	− 0.526	0.59	0.197	0.008	0.40	0.86
	Being depressed	− 0.400	0.67	0.138	0.004	0.51	0.87
Follow-up after 1 month	Use heated tobacco product	− 0.020	0.98	0.225	0.931	0.63	1.52
	Use e-cigarettes	0.077	1.08	0.136	0.569	0.82	1.41
	Use nicotine medications	− 0.035	0.96	0.199	0.861	0.65	1.42
	Current use of any smoking cessation app	− 0.207	0.81	0.165	0.210	0.58	1.12
	Intention to quit smoking	0.048	1.04	0.118	0.687	0.83	1.32
	Perceived helpfulness of the app	0.302	1.35	0.091	< 0.001	1.13	1.61
MODEL2: **Covariates measured at 6 months**							
	Use heated tobacco	0.711	2.03	0.255	0.005	1.23	3.35
	Use e-cigarettes	− 0.054	0.94	0.114	0.634	0.75	1.18
	Use nicotine medications	0.559	1.74	0.194	0.004	1.19	2.55
	Current use of any smoking cessation app	− 0.284	0.75	0.153	0.063	0.55	1.01
	Perceived helpfulness of the app	0.240	0.78	0.082	0.004	0.66	0.92
	Number of different days when the app was accessed (automatically collected)	0.011	1.01	0.004	0.009	1.00	1.02
	Number of times the app was opened (automatically collected)	0.000	1.00	0.001	0.948	0.99	1.00
	App’s use duration (automatically collected)	0.002	1.00	0.001	0.040	1.00	1.01

*b* = beta coefficient; OR = odds-ratio; SE = standard error; *p* = p-value; CI = confidence interval

* = significant at least at *p* < .05

To answer question 3 (i.e. predictors of change in cigarette count/day), we built a machine learning regression model using the RF algorithm [11, 12], with the following setting parameters: “ntree” = 500, meaning that each random forests model was constructed from 500 regression trees, and we left “mtry” - the number of predictors available for allocation at each tree node - at the default value of one third of the total number of predictors. RF regression models provide, among other results, the significance of each predictor based on a measure called %IncMSE (percentage increase in mean squared error). The %IncMSE expresses the increase in MSE (estimated with out-of-bag cross-validation) as a result of the permutation of variable j (randomly shuffled values); in simple terms, it describes how much (in percent) the MSE increases by excluding each variable. The more the MSE increases, the more important the variable is for successful prediction. The variables can thus be ranked in order of importance (Table 4).

**Table 4:** Predictors of change in cigarettes per day after 6 months, in decreasing order of importance

Predictors measured at baseline or after 1 month	%IncMSE
Cigarettes/day as measured at baseline	46.11
Use of nicotine medications after one month	12.49
Use of e-cigarettes as self-reported after one month	8.25
Intention to quit smoking as measured after one month	7.65
Number of different days when the app was opened (automatically collected)	6.53
Number of times the app was opened (automatically collected)	6.39
Minutes before the first cigarette of the day	4.27
Current use of any smoking cessation app after one month	3.91
App’s use duration (automatically collected)	3.85
Perceived helpfulness of the app after one month	3.71
Age	3.01
Used of heated tobacco at baseline	3.01
Number of years smoking	2.98
Used of heated tobacco product at one month	2.69
Sex	2.33
Smoking other tobacco products at baseline	1.22
Use of nicotine medications at baseline	1.13
Use of e-cigarettes at baseline	1.05
Being depressed	0.23
Country (Switzerland vs. France)	0.09
Experiment group	0.02

%InMSE = per cent increase in mean squared error, a statistical measure indicating the level on the predictor variable importance in the regression machine learning algorithm

To answer question 4 (relationships between each predictor and covariate and change in cigarette count/day), we first wanted to build multiple linear regression models for the continuous/ordered variables and ANOVA models for the nominal independent variables. Since the outcome variable did not meet the normality assumption (Shapiro-Wilk test = 958; p < .001), we had to use a non-parametric regression model instead. We therefore applied generalised additive modelling (GAM) [13–15] using the “mgcv” package for R. We then selected the best model based on the R^2^ and AIC metrics. The selected model had the following tuning parameters: function = gam, method = REML, family = exponential, k = 3, select = TRUE, optimizer = newton. In addition to the GAM model (Table 5), we ran an ANOVA test (using SPSS software version 28.0) after removing a dozen outliers from the dataset and normalising the outcome variable (Table 6).

**Table 5:** Effects of continuous and categorical-ordered predictors and covariates on change in cigarettes/day between baseline and 6 months: summary of the GAM model

Predictors’ category	Predictors	Min-Max	M	SD	Edf	Ref.df	*F*	*p*
Demographics	Age	19–75	38.57	10.81	1	1	0.98	0.344
Baseline	Number of years smoking	1–56	19.60	11.16	1	1.81	12.05	0.019
	Cig./day	1–60	15.25	7.40	1	1.98	28.19	< 0.001
	min before the first cigarette of the day	5-1080	19.29	11.19	1	1.96	15.25	0.001
	Use other tobacco products	1–4	1.86	1.12	1	1	0.85	0.391
	Use e-cigarettes	1–4	1.75	1.09	1	1	1.31	0.227
Follow-up at 1 month	Use e-cigarettes	1–4	1.45	0.80	1	1	1.14	0.616
	Intention to quit smoking	1–3	1.44	0.57	1	1.96	14.26	< 0.001*
	Perceived helpfulness of the app	1–6	3.30	1.17	1	1	2.12	0.072
Follow-up at 6 months	Use of e-cigarettes	1–3	1,54	0.86	1	1	0.42	0.611
	Perceived helpfulness of the app	1–6	3.40	1.14	1	1.98	24.33	< 0.001*
	Number of different days when the app was accessed (automatically collected)	1-341	23.52	34.67	1	1	0.26	0.751
	Number of times the app was opened (automatically collected)	1-5100	111.22	232.74	1	1	0.52	0.110
	App’s use duration (automatically collected)	0-498	113.36	117.38	1.543	1.79	0.63	0.585

Min = minimum, Max = maximum, M = mean, SD = standard deviation; edf = effective degrees of freedom; Ref.df = reference degrees of freedom used on computing test statistic and the p-values; *F* = variance ratio; *p* = p-value

* = significant at *p* < .05 or p < .001

The change in cigarettes/day between baseline and 6 months (outcome variables) descriptive statistics: Min/Max (18/-50); M = -6.95; SD = 7.46.

**Table 6:** Effects of nominal predictors and covariates on change in cigarettes/day between baseline and 6 months: one-way ANOVA

Predictors’ category	Predictors	Yes (M/SD)	No (M/SD)	*F*	*p*
Demographics	Country	France: -6.87/7.56	Switzerland: -7.12/7.23	0.327	0.568
	Sex	Male: -8.87/8.37	Female: -5.99/6.77	41.386	< 0.001*
Treat/Control	Experiment group	Experiment: -7.05/7.59	Control: -6.86/7.35	0.231	0.631
Baseline	Use heated tobacco	-7.47/5.70	-6.95/7.51	0.324	0.572
	Use nicotine medications	-9.11/7.30	-6.43/7.41	28.844	< 0.001*
	Being depressed	-7.26/7.69	-6.50/7.10	3.560	0.059
Follow-up at 1 month	Use heated tobacco	-9.20/8.36	-7.04/7.41	0.032	0.859
	Use nicotine medications	-9.37/7.99	-6.79/7.22	102.001	< 0.001*
	Current use of any smoking cessation app	-7.47/7.22	-6.79/7.71	16.654	< 0.001*
Follow-up at 6 months	Use heated tobacco	-6.84/7.35	-6.97/7.48	0.08	0.892
	Use of nicotine medications	-8.26/8.02	-6.28/7.07	21.173	< 0.001*
	Current use of any smoking cessation app	-7.34/7.80	-6.54/7.08	3.755	0.069

M = Mean; SD = Standard deviation. Degrees of freedom 1 = 1 for all analyses; degrees of freedom 2 = 871 for all analyses. *F* = variance ratio; *p* = p-value

* = significant at *p* < .05 or p < .001

In this table, the negative sign (-) preceding the mean values just means “reduction”. Thus, the mean values presented in this table must be consider as “the mean of the number of cigarettes/days reduction”

To answer question 5 (i.e. predictors of smoking relapse), we built a machine learning model using the RF classification algorithm [11, 12] (Table 7).

To answer question 6 (i.e. relationship between each predictor and relapse to smoking and association between each covariate and smoking recidivism), webuilt a multinomial logistic regression model using SPSS version 28.0 (Table 8).

Some of follow-up measures had missing values. For all random forest analyses, the missing values were handled with the “rfImpute()” function of the RF algorithm, which uses a nearest-neighbour machine learning approach to either impute values or weight their absence [11, 12]. The dataset used for the regression analysis and for ANOVA had the missing data imputed also by the “rfImpute()” RF function. In addition, for each analysis, we controlled whether imputation had an impact on the analysis results, which it did not.

Finally, we used the RF machine learning algorithm instead of traditional methods because this algorithm is one of the most efficient tools for imputing missing data, has parameters that can be adjusted to improve specific performance metrics, and thus creates a model that best fits the data [11, 12]. However, machine learning algorithms are usually designed to make predictions but do not provide inferential statistics. For this reason, we resorted to traditional methods (regression and ANOVA) to obtain inferential information (metrics for the probability of association between variables). In sum, we chose RF because it offers the possibility of imputing missing data, because it is one of the best existing machine learning algorithms for prediction [11, 12] and because it ranks the most important predictors. We used RF modelling to check for multicollinearity, then we used conventional regression algorithms to perform inferential analysis, and finally we drew conclusions from an interpretation of these analyses.

## Results

### Characteristics of participants

At baseline, the 5293 daily smokers who participated in the randomised trial were on average 38.6 years old (range 19–75 years, SD = 10.8), most were women (67%), most lived in France (71%), and most (59%) had a positive depression screening test. Participants smoked an average of 15.8 cigarettes per day (SD = 7.6), they smoked the first cigarette of the day on average 50 minutes (SD = 96) after waking up, and they had smoked for 17 years (SD = 10.7). Most (n = 871, 62%) of the 1407 participants who completed the 6-month follow-up questionnaire were still smoking after 6 months. Of these 871 participants, 673 reported their number of cigarettes/day both at baseline and after 6 months. They smoked an average of 9.9 cigarettes/day after 6 months (SD = 7.3), and 479 of them (71%) decreased the number of cigarettes/day, 118 (18%) did not change it, and 76 (11%) increased it between the start of the study and the 6-month survey. Of the 502 who responded after 6 months and had stopped smoking after 1 month (criterion: not smoking in the last 7 days), 147 (29%) had relapsed after 6 months and 355 (71%) were still not smoking (criterion: not smoking in the last 4 weeks at 6 months).

Intervention group members liked the app more and used it more often than control group members: over a 6-month period, they used the app an average of 81 vs. 37 times, on 15 vs. 9 different days, and over a period of 75 vs. 55 days (all p < 0.001). Smoking cessation rates at follow-up were similar in the Stop-Tabac group and the control group [7].

Compared with non-respondents to the 6-month survey, respondents were 5 years older, more likely to live in Switzerland (vs France), less likely to be depressed, and they were slightly less dependent on cigarettes. Respondents used the app about 3 times more often than non-respondents, and they used it during 2 more months (Table 1).

### The best predictors of smoking cessation after six months

Table 2 presents the 22 best predictors of smoking cessation after 6 months, in decreasing order of importance, it is based on a machine learning model with 1407 participants.

The top 10 predictors of smoking cessation were, in this order: the number of different days the app was opened; the level of intention to quit smoking measured after one month; duration of app use; number of times the app was opened; cigarettes/day measured at baseline; self-reported current use of a smoking cessation app after one month; perceived usefulness of the app after one month; age; heated tobacco use at baseline; and number of years of smoking.

The value associated with MDA (e.g. MDA = 20.22 for the number of different days the app was opened) means that the model loses this value (20.22 points) in overall accuracy when the corresponding predictor variable is removed.

### Associations between predictors / covariates and smoking cessation after six months

Table 3 shows the results of two different multinomial logistic regression models assessing the relationships between each predictor measured at baseline or after one month (model 1) and the outcome (smoking cessation at 6 months), or each covariate measured at 6 months and smoking cessation at 6 months (model 2). As a reminder, the group of participants who quit smoking was labelled “1” and the group of those who did not quit smoking was labelled “0”. Class “0” was set as the reference class so that our models predict the probability that a person belongs to class “1”.

As shown in Table 3, a higher number of years of smoking was weakly associated with a lower probability of quitting smoking after six months (OR = 0.97 per year); a higher number of minutes before the first cigarette of the day (i.e., lower dependence) was associated with a lower probability of quitting smoking after six months (OR = 0.77 per minute, i.e. a longer time before the first cigarette [i.e. lower dependence] decreased the odds of quitting); participants who smoked other tobacco products at baseline were more likely to quit smoking after six months (OR = 1.25 per point on a 4-point scale); depressed participants were 33% less likely to quit smoking after six months than non-depressed individuals (OR = 0.67); and higher levels of perceived helpfulness of the app increased the likelihood of quitting smoking after six months by 35% per point (OR = 1.35 per point on a 6-point scale).

Among the correlates measured after 6 months: using heated tobacco at 6 months was associated with being an ex-smoker at 6 months (OR = 2.03); using nicotine medication at 6 months was associated with quitting smoking at 6 months (users vs. non-users: OR = 1.74); and a higher number of different days using the app was associated with quitting smoking at 6 months by 1% per day (OR = 1.01 per day).

### The best predictors of change over time in cig./day

Table 4 displays the 22 best predictors of change in cig/day between baseline and 6 months in those who continued to smoke, in decreasing order of importance, it is based on a machine learning model with 673 smokers who answered the questions on cig./day on both time points.

The top 10 predictors of change over time in cigarettes/day were, in this order: cigarettes/day at baseline, use of nicotine medication after one month, use of e-cigarettes after one month, intention to quit smoking measured after one month, number of different days the app was opened, number of times the app was opened, number of minutes before smoking the first cigarette of the day measured at baseline, self-reported current use of a smoking cessation app after one month, and duration of app use.

The value associated with the %IncMSE (e.g. 46.11) means that the RMSE increases by 46.11% when the corresponding predictor variable is removed (in this case, cigarette/day measured at baseline).

### The effects of predictors on change in cig./day over time

Tables 5 and 6 show the effects of the independent variables on the change in cigarette consumption after 6 months among those who continued to smoke, a summary of the GAM model (Table 5, in terms of the continuous and categorical predictors or covariates) and ANOVA (Table 6, in terms of the nominal predictors or covariates).

In Tables 5 and 6, the predictors (i.e. variables measured at baseline or after one month) that had a significant effect on the change in the cigarettes/day within six months were as follows: male participants were more likely to reduce cig./day than female participants; participants who had smoked for more years were more likely to reduce cig./day; participants who had smoked more cigarettes at baseline were more likely to reduce cig./day, participants who had smoked more cigarettes per day at baseline were more likely to decrease cig./day over time; participants who had smoked their first cigarette later in the day (i.e. who were less dependent) were more likely to increase cig./day over time; e-cigarette users were more likely to decrease cig./day than non-users; participants who were taking nicotine medications at baseline or after one month were more likely to decrease cig./day than non-users.

*Six-month follow-up: associations with reduction in cig./day*:

Participants who found the app very helpful after six months were more likely to reduce cigarettes/day; participants who took nicotine-containing medications after six months were more likely to reduce cigarettes/day than participants who did not take these medications; and participants who found the app helpful after six months were more likely to reduce cigarettes/day.

### The best predictors of relapse at six months

Table 7 (machine learning model with 502 participants) presents the 22 best predictors of smoking relapse after six months in those who had quit at one month, in decreasing order of importance.

**Table 7:** Predictors of smoking relapse after six months, in decreasing order of importance

Predictors	MDA
Number of different days when the app was accessed (automatically collected)	17.13
Intention to quit smoking assessed after one month	10.77
Number of times the app was opened (automatically collected)	8.98
Perceived helpfulness of the app measured after one month	8.32
Current use of any smoking cessation app, as self-reported at one month	6.03
Use of nicotine medications at baseline	4.66
Cigarettes/day measured at baseline	3.58
Minutes before the first cigarette of the day	2.12
Use of e-cigarettes as self-reported after 1 month	1.85
Interval (in days) between first and last time they used app (automatically collected)	1.73
Use of e-cigarette et baseline	1.57
Sex	1.48
Age	1.39
Number of years smoking	1.24
Use of nicotine medications at one month	1.03
Being depressed	0.95
Use heated tobacco at baseline	0.68
Use/smoke other tobacco products at baseline	0.37
Experimental group	0.29
Use heated tobacco at one month	0.08

MDA = mean decrease accuracy, a statistical measure indicating the level on the predictor variable importance in the classification machine learning algorithm

The top 10 predictors of smoking relapse between months 1 and 6 were, in this order: the number of different days the app was accessed; intention to quit smoking assessed after one month; number of times the app was opened; perceived usefulness of the app measured after one month; self-report of current use of a smoking cessation app after one month; nicotine medication use at baseline; number of cigarettes/day measured at baseline; number of minutes before first cigarette of the day; e-cigarette use after one month; and duration of app use.

### Associations between predictors / covariates and smoking relapse after six months

Table 8 (model with 502 participants) shows the results of two different multinomial logistic regression models assessing the relationships between each predictor measured at baseline or after one month (model 1) and the outcome (smoking relapse at 6 months), or each covariate measured at 6 months and smoking relapse at 6 months (model 2).

**Table 8:** Predictors and covariates of smoking relapse after six months, from two multivariate logistic regression models

Predictors’ category	Predictors / covariates	*b*	OR	SE	*p*	95%CI on OR	
MODEL 1: **Predictors measured at baseline and after 1 month**							
Demographics	Country	− 0.263	0.76	0.263	0.317	0.45	1.28
	Age	− 0.008	0.99	0.029	0.793	0.93	1.05
	Sex	− 0.025	0.97	0.234	0.915	0.61	1.54
Treat/Control	Experiment group	− 0.336	0.71	0.242	0.165	0.44	1.14
Baseline	Number of years smoking	− 0.031	0.97	0.015	0.038	0.94	0.99
	Cigarettes/day	0.003	1.00	0.017	0.873	0.97	1.03
	min before the first cigarette of the day	− 0.003	0.99	0.001	0.045	0.99	1.00
	Use other tobacco products	− 0.160	0.85	0.094	0.090	0.70	1.02
	Use heated tobacco	− 0.614	0.54	0.618	0.321	0.16	1.81
	Use e-cigarettes	0.118	1.12	0.123	0.337	0.88	1.43
	Use nicotine medications	− 0.864	0.42	0.316	0.006	0.22	0.78
	Being depressed	− 0.195	0.82	0.215	0.363	0.54	1.25
Follow-up after 1 month	Used heated tobacco	1.404	4.07	0.807	0.082	0.83	19.78
	Use e-cigarettes	− 0.239	0.78	0.201	0.234	0.53	1.16
	Use nicotine medications	− 0.029	0.97	0.317	0.927	0.52	1.80
	Current use of any smoking cessation app	− 0.612	0.54	0.252	0.015	0.33	0.88
	Intention to quit smoking	-1.278	0.27	0.351	< 0.001	0.14	0.55
	Perceived helpfulness of the app	− 0.270	0.76	0.123	0.028	0.60	0.97
MODEL2: **Covariates measured at 6 months**							
	Used heated tobacco	− 0.057	0.94	0.335	0.865	0.48	1.82
	Use e-cigarettes	0.017	1.01	0.194	0.931	0.69	1.48
	Use nicotine medication	0.354	1.42	0.306	0.247	0.78	2.59
	Current use of any smoking cessation app	0.072	1.07	0.244	0.767	0.66	1.73
	Perceived helpfulness of the app	− 0.333	0.71	0.131	0.011	0.55	0.92
	Number of different days when the app was accessed (automatically collected)	0.004	1.00	0.009	0.647	0.98	1.02
	Number of times the app was opened (automatically collected)	0.002	1.00	0.002	0.226	0.99	1.00
	App’s use duration (automatically collected)	− 0.002	0.99	0.001	0.128	0.99	1.00

*b* = beta coefficient; OR = odds-ratio; SE = standard error; *p* = p-value; CI = confidence interval

As shown in Table 8, the more years participants had smoked (OR = 0.97 per year), the less likely they were to relapse; the longer the interval between waking up and smoking the first cigarette of the day (i.e., the lower the dependence), the less likely they were to relapse (OR = 0.99 per minute); participants who were taking nicotine medication at baseline were less likely to relapse (OR = 0.42 compared to non-users); participants who reported using a smoking cessation app after one month were less likely to relapse (OR = 0.54); participants who intended to quit smoking after one month were less likely to relapse (OR = 0.27 per point on a 3-point scale); and finally, participants who found the app more helpful were less likely to relapse (OR = 0.76 after one month, OR = 0.71 after 6 months per point on a 6-point scale).

## Discussion

Among users of a smartphone app for smoking cessation who participated online in a randomised trial testing the effectiveness of this app, smoking cessation after 6 months was predicted primarily by the degree of tobacco dependence (cigarettes/day and minutes to first cigarette), by the degree of motivation to quit smoking, by the frequency and duration of use of the app and its perceived usefulness, and by the use of nicotine medication.

Among those who continued to smoke after 6 months, reduction in the number of cigarettes/day was mainly predicted by level of tobacco dependence (cigarettes/day and minutes to first cigarette), use of nicotine medication, frequency of app use and its perceived usefulness, and use of e-cigarettes. The reduction in the number of cigarettes per day over time was greatest among those who had smoked more cigarettes per day at baseline, but this may reflect a phenomenon of regression to the mean [16], as there were no multiple assessments before the intervention, it is difficult to completely rule out such a phenomenon [17, 18].

Among those who had quit smoking after one month, relapse to smoking after six months was mainly predicted by intention to quit, frequency and intensity of app use, perceived usefulness of the app, level of dependence and use of nicotine medication.

Being in the experimental group (compared to the control group) was not a predictor of smoking status or smoking reduction at 6 months, but it did increase the likelihood of nicotine medication use, as previously reported [7].

The importance of tobacco dependence, motivation to quit smoking, and nicotine therapy as predictors of smoking cessation, smoking reduction and relapse is well documented [4–6, 19]. This study provides new insights by showing strong associations between the level of engagement with the app (frequency and duration of use, perceived usefulness) and outcomes, after statistical adjustment for other predictors. More frequent use of the app was associated with a higher likelihood of quitting or cutting down on smoking and a lower likelihood of relapse, in multivariate analyses adjusting for other factors. App use patterns and perceived helpfulness could be considered as proxies for the digital therapeutic alliance, which was previously shown to be associated with increased engagement with smartphone apps and appears to influence outcomes [20–22].

The causality can go both ways: either more frequent use of the app increased the likelihood of quitting or reducing smoking, or those who quit had withdrawal symptoms and therefore needed more support and consequently used the app more often. According to the Self-determination theory, participants might also need and possibly found more support in the app, in the form of relationships (discussion forum) and behaviour change skills, regardless of withdrawal symptoms [23, 24, 25].

In addition, both smoking cessation/reduction and app use may be influenced by other factors (e.g. motivation, social support and social pressure, environmental factors) that may contribute to the present results.

The association between frequency of app use and smoking cessation or reduction was also observed among control app users, either supporting the latter two explanations or suggesting that the control app was effective despite its limited content.

Although these correlational analyses cannot prove causality, they can help us formulate hypotheses that can later be tested experimentally. For example, the effectiveness of the app could be improved if it included additional features that increased the frequency or duration of use, and user satisfaction, e.g. by expanding the existing social network within the app to make it more active or supportive (the user would then open the app more frequently to read or post messages and receive encouragement from peers), or by further developing the existing customised feedback support system to make it more intensive or more timely (e.g., ensuring that automated, individualised messages are available during a relapse episode or during episodes of craving and other withdrawal symptoms), or the app could also be improved if it further increased participants’ use of nicotine medications, or if it increased their use of external supports such as smoking cessation services and local clinics.

The association between heated tobacco use and smoking cessation after adjustment for other predictors yields a hypothesis that should be tested experimentally [26].

Incidentally, most of the study participants tested positive for depression, and depressed participants were less likely to quit smoking after six months than non-depressed participants. This result is consistent with other studies showing that patients who come to a smoking cessation clinic with depressive symptoms are less likely to quit smoking than patients without such symptoms [27]. This may argue for adding psychosocial mood management components to smoking cessation apps to meet the needs of depressed people [28].

Finally, in the current study, men were more likely to reduce their cigarette consumption than women. This finding is consistent with recent studies showing that withdrawal symptoms are more pronounced in female smokers than in male smokers and that female smokers are more likely to relapse than male smokers [29]; all of which suggests that it may be more difficult for women to quit smoking than for men.

### Limitations and strengths

As this study was not originally intended for the present secondary data analysis, the dataset included only a limited number of predictor variables, which limited our ability to predict smoking cessation, reduction and relapse. The response rate at 6-month follow-up was low (27%), and respondents to the 6-month survey differed from non-respondents (respondents were older, less depressed, they used the app more frequently), and this may limit he generalizability of our results. The low response rate at follow-up can possibly be explained by the fact that the participants had never met the researchers personally and therefore may not have felt connected to them.

Strengths of the study include a large sample of people of all ages who lived in all departments of France and all cantons of French-speaking Switzerland, and the collection of data on app use from 100% of participants. Finally, our results may not be transferable to users of other smoking cessation apps.

## Conclusion

In addition to well-documented predictors of smoking cessation, reduction, and relapse (e.g., level of dependence, motivation to quit, use of nicotine medication) [4–6], this study showed that smoking cessation, reduction, and relapse were predicted by frequency and duration of app use and perceived usefulness of the app, after adjusting for other predictors in multivariate models. Studies on the predictors of smoking behavior among users of smoking cessation apps may provide useful insights for the future development of these apps and future experimental studies.

## Data Availability

The questionnaires used in this study are available at: https://archive.org/details/@stopdependance_ch. The original data are available in open sources at Yareta: https://yareta.unige.ch/home/detail/d8d9b746-3fe4-4077-a4f3-552154c16699.

## List of abbreviations

apps: Application
cig: Cigarettes
RQ: Research question.
MDA: Mean Decrease Accuracy.
%InMSE: Per cent increase in mean squared error,
OR: Odds-ratio.
ANOVA: Analysis of variance
